# Development of Immunotherapy Strategies Targeting Tumor Microenvironment Is Fiercely Ongoing

**DOI:** 10.3389/fimmu.2022.890166

**Published:** 2022-06-27

**Authors:** Rilan Bai, Jiuwei Cui

**Affiliations:** Cancer Center, The First Hospital of Jilin University, Changchun, China

**Keywords:** cancer, tumor microenvironment, immunotherapy, inhibitory signaling, stimulatory signaling

## Abstract

Tumor immune microenvironment is a very complex system that is influenced by a wide range of factors; in this microenvironment, various immune cells, stromal cells, and cytokines can interact with tumor cells and jointly regulate this complex ecosystem. During tumor development, the tumor microenvironment (TME) shows the upregulation of inhibitory signals and downregulation of activating signals, which result in an immunosuppressive microenvironment and lead to tumor immune escape. In recent years, a variety of precision immunotherapy strategies have been developed to remodel the TME into a positive immune microenvironment by stimulating or restoring the inherent tumor inhibition ability of the immune system so as to improve anti-tumor therapeutic efficacy. This review focuses on immunotherapy strategies targeting the TME, including those that target the microenvironment to inhibit signaling, activate signaling, and specifically involve many new targets such as physical barriers, immune cells and their surface molecular receptors, cytokines, and metabolic factors. Furthermore, it summarizes the challenges faced while conducting research on the tumor immune microenvironment and the corresponding solutions.

## 1 Introduction

The tumor immune microenvironment (TIME) is a very complex system that is influenced by a wide range of factors; in this microenvironment, various immune cells, stromal cells, and cytokines can interact with tumor cells. The regulation of these immune system networks and the complex interaction between tumors can have an important impact on tumor development and immunotherapy response: “what happens to a small part may affect the whole.” Tumorous tissues depend on the microenvironment for survival and jointly regulate complex ecosystems. Many factors within a tumor can affect and induce a tumor microenvironment (TME), which in turn can promote tumor development and affect the efficacy of anti-tumor therapy. Tumor-associated macrophages, tumor-associated fibroblasts, and mesenchymal stem cells can enhance tumor drug resistance by recruiting and secreting a variety of protective cytokines. Non-cellular components such as extracellular matrix and conditions such as hypoxia and acidification can mediate resistance to anti-tumor therapy by constructing a physical barrier and affecting the growth and metabolism of tumor cells. In the process of tumor development, the TME shows the upregulation of inhibitory signals and downregulation of activating signals, which result in an immunosuppressive microenvironment. It is closely related to both tumor immune escape and anti-tumor treatment efficacy. Therefore, immunotherapeutic strategies targeting the TME can stimulate or restore the inherent tumor suppression ability of the immune system, remodel it into a positive immune microenvironment, and yield comprehensive response effects. In recent years, with the development and continuous improvement of multiplex immunohistochemical technology, high-throughput sequencing, and microarray technology, the understanding and cognition of TME factors have gradually deepened, and a variety of precision immunotherapy strategies targeting TME have been developed. This review focuses on immunotherapy strategies targeting the TME and summarizes the challenges faced while conducting research of on the TIME and the corresponding solutions.

## 2 Immunotherapeutic Strategies Targeting the TME

### 2.1 Therapeutic Strategies Based on TME Inhibitory Signaling

TME is characterized by a variety of inhibitory signals, including TME physical barriers, inhibitory immune cells and their surface inhibitory receptor signals, and metabolic inhibitory signals, which promote tumor development and immune escape and affect the efficacy of and resistance to anti-tumor immunotherapy. Strategies targeting these inhibitory signals can reverse T-cell depletion and the overall state of the inhibitory immune microenvironment, contribute to the normalization of the immune microenvironment, restore the tumor-suppressive ability of the immune system, and significantly improve anti-tumor immunotherapy efficacy.

#### 2.1.1 Targeting TME Physical Barriers

*In vivo*, cancer cells are located in a complex 3D microenvironment, and its physical barriers, including the extracellular matrix, fibroblast activating protein (FAP), collagen, and laminin (FAK), are considered great obstacles for cancer therapy (1). The rapid growth of tumors disrupts the structure and function of surrounding tissues and leads to the presentation of unique physical cues of the TME, such as increased matrix stiffness, changes in vascular shear stress, and changes in the extracellular matrix (ECM) structure (2), which affect the biological behavior of cancer cells and lead to the formation of a heterogeneous immunophenotype of TMEs by interfering with the integrity of the cancer-immune cycle, promoting tumor progression further, and affecting tumor responsiveness to immunotherapy (2–5). Therefore, overcoming the physical barriers in the TME may improve the efficacy of immunotherapy. In recent years, a variety of strategies and drugs have been developed to reverse the immunophenotype of TME: these include inhibition of Rho-kinase and FAK-mediated cell contraction (e.g., Fasudi, H1152, Defactintb), reduction of matrix components (e.g., FAP gene-editing cells, FAP-vaccine, FAP antibody-nanoparticle, VS-4718, and PEGPH20), inhibition of matrix cross-linking (e.g., BAPN and miRNA LOX inhibitors) and fibrosis (e.g., pirfenidone, losartan, and tranilast), improvement of tumor vascular leakage (e.g., bevacizumab), and reduction of the effect of vascular shear stress (2, 6, 7).

Vascular abnormalities are hallmarks of most solid tumors, and they mediate immunosuppressive microenvironments and immune evasion. The rational use of drugs that target these molecules can improve therapeutic responsiveness, partially because abnormal tumor vasculature returns to normal, and anti-angiogenic drugs increase the infiltration of immune effector cells into tumors and transform the intrinsic immunosuppressive TME into an immunosupportive one (8). Therefore, the combination of anti-angiogenic therapy and immunotherapy may have synergistic effects and reduce the risk of immune-related adverse events. The IMpower 150 study (9) showed that the combination with atezolizumab, an immunotherapeutic agent, with platinum-based chemotherapy + anti-angiogenic therapeutic agents can exert a new synergistic effect. The LEAP-006 (NCT03829319) study was a phase 3, double-blind, randomized clinical study that used pembrolizumab in combination with pemetrexed and platinum-based chemotherapy ± lenvatinib in patients with advanced first-line non-squamous non-small-cell lung cancer (NSCLC). The LEAP-007 study (NCT03829332) was a phase 3, double-blind, randomized clinical study that used pembrolizumab alone or in combination with lenvatinib in patients with advanced first-line NSCLC with programmed death ligand 1 (PD-L1) tumor proportion score (TPS) ≥ 1%; this study explored the synergistic effect of anti-angiogenic therapy with other immunotherapies. In the future, emerging innovative technologies, such as cancer microarrays, immuno-engineering technologies, cancer mathematical models, and deep machine learning, can comprehensively explore the characteristics of TME physics and use this as a basis to develop immunotherapeutic strategies targeting TME physical barriers.

#### 2.1.2 Targeting Immune Checkpoints

Immune checkpoint inhibitors targeting cytotoxic T lymphocyte-associated antigen-4 (CTLA-4) (ipilimumab) and programmed cell death-1/PD-L1 (PD-1/PD-L1) (nivolumab, pembrolizumab, atezolizumab, avelumab and durvalumab) have been found to be effective against many types of tumors (10–12) and approved by the Food Drug Administration (FDA) for the treatment of a variety of tumors. In recent years, novel checkpoint molecules such as lymphocyte-activation gene-3 (LAG-3), T-cell immunoglobulin 3 (TIM-3), and T-cell immunoglobulin and ITIM domain (TIGIT) have been widely and intensively studied in terms of the tumor immunosuppressive microenvironment, “T-cell depletion,” and corresponding targeted antibody therapy (13). LAG-3, a type I transmembrane protein that is mainly expressed in activated T cells, natural killer cells (NKs), B cells, and plasmacytoid dendritic cells (DCs), is involved in the transduction of immune cell inhibitory signals (14). Currently available drugs for LAG-3 include relatlimab (BMS-986916), LAG525 (IMP701), MK-4280, TSR-033, REGN3767, Sym022, INCAGN02385, FS118, BI754111, and MGD013. The global, randomized, double-blind, phase II/III RELATIVITY-047 (CA224-047) trial is the first first-line treatment study to demonstrate that dual inhibitory effects of LAG-3, and the PD-1 pathways may be a key target to enhance the immune response and help improve the prognosis of patients with metastatic or unresectable melanoma (15). Eftilagimod alpha (Efti, IMP321, or LAG-3Ig) is a soluble LAG-3 fusion protein composed of the extracellular domain of LAG-3 and the Fc region of IgG that may activate antigen-presenting cells (DCs) through major histocompatibility complex (MHC)-II-mediated signaling; this activation results in an increase in interleukin (IL)-12 and tumor necrosis factor (TNF) levels and upregulation of CD80 and CD86 expression, along with the removal of the inhibitory effect of DCs on T cells through LAG-3 (16–18). The phase III TACTI-002 clinical trial (NCT03625323) used Eftilagimod alpha in combination with pembrolizumab as the first-line treatment for advanced tumors and metastatic NSCLC (part A) (19) and as second-line treatment for NSCLC (refractory to PD-1/PD-L1) (part B) and metastatic head and neck cancer (platinum-resistant) (part C) (20). Based on preliminary efficacy data, the FDA awarded the soluble LAG-3 protein eftilagimod alpha fast-track designation in April 2021; the phase II TACTI-003 study was conducted on LAG-3Ig combined with pembrolizumab as the first-line treatment for head and neck squamous cell carcinoma, while the INSIGHT-004 study is an ongoing study on LAG-3Ig combined with avelumab for the treatment of a variety of advanced solid tumors.

Interaction of TIM-3 with its ligand galectin-9 (Gal-9) inhibits the activity of T cells, prompts them to present a “depletion phenomenon,” and regulates apoptosis and immune tolerance of T cells (21, 22). Blocking the pathway of TIM-3/Gal-9 binding may break the “depletion phenomenon” of T cells, and a combination of two monoclonal antibodies, anti-PD-1 and anti-TIM-3, can synergistically break the drug-resistant TIME, which is a promising novel cancer immunotherapy regimen (23). At present, several phase I/II clinical trials are ongoing with anti-TIM-3 or its combination or anti-TIM-3/PD-1/L1 bispecific antibodies (NCT03489343, NCT03652077, NCT03099109, NCT02608268, etc.). For TIGIT target, the ongoing/upcoming phase III clinical studies on advanced first-line NSCLC treatment in China include the following: MK-7684A-003 trial (PD-L1 ≥ 1%, MK-7684 + Pembrolizumab, NCT04738487), AdvanTIG-302 trial (PD-L1 ≥ 50%, BGB-A1217 + Teicilizumab, NCT04746924), and SKYSCRAPER-01 trial (high PD-L1 expression, Tiragolumab + Atenibizumab, NCT04294810). An *in vitro* cell interaction analysis system confirmed that Siglec-15 (S15) was highly expressed in macrophages, and Siglec/sialyloglycan axis activation could directly inhibit T-cell activity and play an important role in the process of immune escape in tumor cells (24). The NC318 monoclonal antibody against Siglec-15 is currently undergoing clinical studies (25). It is worth mentioning that the expression of Siglec-15 does not affect that of PD-L1 or vice versa, which provides a new strategy for the treatment of patients who develop resistance to PD-1/PD-L1 or patients with very low PD-L1 expression (24).

#### 2.1.3 Targeting Immunosuppressive Cells

Major factors hindering the function of effector T cells in the TME are immunosuppressive myeloid and lymphoid cells, including myeloid-derived suppressor cells (MDSCs), immunosuppressive macrophages, regulatory cells (Tregs), and immature DCs, which can promote tumor immune escape *via* the production of immunosuppressive cytokines. Targeting these suppressive immune cells and reversing their immunosuppressive effects on the microenvironment are effective measures to improve the anti-tumor immune response. M2 macrophages are predominant in the TME, and promoting the reprogramming of M2 macrophages to inhibit the M1 phenotype of tumors is an effective approach for improving the TIME significantly. Tumor cells can regulate the proliferation and differentiation of macrophages to the M2 type with the help of class IIa HDACs (26); thus, TMP195, an HDAC inhibitor, can reduce the number of M2 macrophages in mice and improve the efficacy and tolerability of chemotherapy and PD-1 inhibitors (27). Sitravatinib is an RTK inhibitor that targets tumor-associated macrophage (TAM) receptors (TYRO3, AXL, MerTK) and a variety of similar RTKs, including those associated with angiogenesis (e.g., VEGFR2, KIT), RET, and MET (28), which make sitravatinib important for improving the tumor immunosuppressive microenvironment. Moreover, sitravatinib can transform M2 macrophages with immunosuppressive function into M1 macrophages, increase the number of CD8^+^ T cells, and decrease the number of Treg and MDSCs cells (28). Therefore, sitravatinib can lead to changes in innate and adaptive immune cells, thereby enhancing the immune checkpoint blockade. In an open-label clinical phase II study (MRTX-500) (29), sitravatinib in combination with nivolumab showed good clinical efficacy in patients with non-squamous NSCLC whose disease progressed on previous anti-PD-1/L1 regimen, with a primary endpoint objective response rate (ORR) of 18% (12/68), median progression-free-survival (mPFS) of 5.7 months, and median overall survival (mOS) of 14.9 months. Sitravatinib in combination with nivolumab showed better anti-tumor activity and OS than did the control in previous studies, with no new safety signals observed. On the basis of this finding, a global multicenter phase III SAPPHIRE study (NCT03906071) is being conducted to further assess the feasibility of this regimen. Currently, sitravatinib is undergoing clinical trials for multiple indications (NCT02978859, NCT02219711, NCT02954991, and NCT03015740). These results highlight the potential immune-activating effects of sitravatinib and the synergistic effects of combination therapy with other immune checkpoint inhibitors (ICIs).

#### 2.1.4 Targeting Inhibitory Cytokines

There are multiple immunosuppressive factors in the TME, and a combination of drugs targeting these immunosuppressive cytokines can effectively improve the efficacy of immunotherapy. Tumors, tumor-associated stromal cells, and Tregs produce large amounts of transforming growth factor-β (TGF-β). TGF-β is a factor the promotes the differentiation of bone marrow and T cells, and it can promote MDSC and Treg differentiation (30). A study showed that the inhibition of TGF-β increases the proliferation and matrix metallopeptidase 9 (MMP-9) expression of tumor-associated fibroblasts (TAF), which negatively regulate PD-L1 expression on the surface of tumor cells and reduce anti-PD-1 efficacy (31). However, this phenomenon can be avoided, and the anti-tumor therapeutic effect can be enhanced when anti-PD-1 and TGF-β inhibitors are used sequentially rather than simultaneously (31). In addition, a bifunctional fusion protein M7824 (Bintrafuspalfa) was developed against PD-L1 and TGF-β, which can improve anti-tumor therapeutic effects by antagonizing PD-L1 and “trapping” TGFβ (32, 33). Colony-stimulating factor-1 (CSF-1) is able promote the function and survival of small glioma cells and TAMs, and the inhibition of the CSF-1 receptor (CSF-1R) relieves TME immunosuppression by depleting TAMs and synergizes with other immunotherapies (34). An open-label, single-arm cohort phase Ia/Ib clinical trial (NCT02526017) that assesses the safety, kinetics, and pharmacodynamics of the CSF-1R antibody cabiralizumab (FPA008) in combination with nivolumab in patients with advanced solid tumors and a phase II clinical trial (NCT03336216) that investigates the efficacy of cabiralizumab and nivolumab with or without chemotherapy in patients with advanced pancreatic cancers are ongoing. Shi et al. (35) combined the CSF-1R inhibitor PLX3397 with oncolytic viruses (OVs) and PD-1 antibodies and showed that CT26 and MC38 subcutaneous xenografts in 43% and 82% of mice, respectively, had complete tumor regression after combination therapy and were able to develop long-term immune memory effects. The combination of these three agents reversed the TME in an immunosuppressive state, increased the infiltration of T cells, reduced the proportion of TAMs, and depleted CD8^+^ T cells. Moreover, the activation level and killing function of T cells increased significantly, which resulted in a strong synergistic anti-tumor effect (35).

#### 2.1.5 Targeting Metabolic Inhibition Signaling

TME metabolism is influenced by many factors, including oncogene-driven intracellular metabolic processes; extracellular factors, such as tissue vascularization, extratumoral nutrition, and oxygen concentration; and other microenvironment-derived factors that determine tumor metabolic characteristics, such as cytokines, hormones, and metabolites that can regulate immune cell metabolism (36). Targeting these metabolic inhibitory molecules or signals in the TME is currently a promising anti-tumor immunotherapeutic strategy. The solute carrier transporter (SLC) family mainly mediates the membrane transport of various solutes and maintains the stability of the intracellular environment. Combined strategies targeting SLCs in the TME can improve antigen presentation and secretion of cytokines, chemokines, and granzymes, thereby improving multiple immune cell functions and mobilizing immune cell-cell interactions. The output of lactate by glycolytic cells and the input of lactate by OXPHOS cells are regulated by the specific transporter monocarboxylate transporter (MCT)-1/4 on the cell membrane, which maintain intercellular lactate metabolic adaptation and symbiosis within tumor tissues and promote tumor growth and metastasis through signaling pathways. At present, multiple small-molecule inhibitors targeting MCT1 have been reported (37), of which AZD3965, a novel drug, is the most striking (38, 39); clinical trials evaluating the effects of this drug on several tumors (NCT01791595). A study that evaluated the effect of nanomedicines composed of MCT1 inhibitors (AZD3965) combined with anti-PD-1 therapy on tumor models reported the potent inhibition of tumor growth and prolongation of survival (40). In addition, the FA receptor CD36 can transport fatty acids into cells and affect tumor cell growth, metastasis, and epithelial-mesenchymal transition (41–43). In tumor mice, an antibody targeting CD36 showed that 15% of metastases (lymph node and lung metastases) achieved a complete response (CR), and mice that had developed lymph node metastases had an 80–90% reduction in lesion size, with little effect on the primary tumor (43). CD36 targeting can also promote a decrease in the number of and apoptosis of intracellular mitochondria in intratumoral Tregs and promote the production of interferon (IFN)-γ^+^ and TNF^+^ CD8^+^ T cells (42). IFN-γ released by CD8^+^ T cells downregulates the expression of SLC3A2 and SLC7A11, which are two subunits of the glutamate-cystine reverse transport system xc^−^, and inhibits cystine uptake in tumor cells (44). Therefore, the combination of ICIs with a synthetic protease that specifically degrades extracellular cystine and cysteine may significantly enhance T-cell-mediated anti-tumor immune responses and induce ferroptosis in tumor cells.

In addition to targeting metabolic receptors, therapeutic strategies target metabolic enzymes within the microenvironment also. Avasimibe is a small-molecule inhibitor that targets acyl coenzyme A cholesterol acyltransferase 1 (ACAT1) in the cholesterol metabolic pathway, which inhibits cholesterol esterification and increases intracellular free cholesterol levels. A study showed that avasimibe can promote pancreatic cancer cell apoptosis by promoting increased ER stress (45), inhibit hepatoma cell proliferation, and improve prognosis (46). A mouse study by Yang et al. (47) confirmed that the inhibition of cholesteryl esterification by gene knockout or ACAT1 inhibitor significantly increased the production of CD3-TCR (T-cell receptor) clusters, effective immune synapses in the T-cell membrane, the proliferation of CD8^+^ tumor infiltrating lymphocyte cells (TILs), and the production of cytolytic granules, cytokines, and their cytotoxic anti-tumor effects; it finally prolonged the inhibition of tumor growth and survival time of mice (47). High indoleamine 2,3-dioxygenase (IDO) expression can lead to local tryptophan depletion in cells, induce T-cell arrest in the G1 phase, inhibit proliferative activation, and strengthen Treg-mediated immunosuppression (48, 49). Although IDO inhibitors can activate T cells in various ways, they do not provide clinical benefits alone. IDO inhibitors can be combined with other anti-cancer drugs, but a trial of their combination with anti-PD-1 has shown disappointing data (50). Interestingly, results of a recent trial that used IO102/IO103, a vaccine under development that targets IDO and PD-L1, in combination with nivolumab for the treatment of metastatic melanoma showed an ORR as high as 80%, with 13 patients (43%) achieving CR (51). In summary, new exploration of TME metabolic patterns should be gradually carried out in the future to study the effect of TME metabolic changes on tumor invasiveness and immune regulation and to develop therapeutic strategies that can target tumor metabolism and activate anti-tumor immunity; this exploration must consider combinations with other metabolic targeted drugs, antibody-based immunotherapy, and tumor vaccines, as this can open up a new horizon for improving the treatment of cancer.

### 2.2 Therapeutic Strategies Based on TME Stimulatory Signals

Activation signals are downregulated in TME, and the treatment strategies targeting this aspect are as follows: activation of stimulatory receptors on the surface of immune cells, supplementation of stimulatory cytokines, enhancement of antigen presentation, and supplementation of immune effector cells, which can reverse the efficacy limitations caused by insufficient immune activation signals in the TME.

#### 2.2.1 Targeting Stimulatory Checkpoints

A variety of stimulatory checkpoint molecules exist on the surface of immune cells in the TME, including CD27, CD40, OX40, glucocorticoid-induced TNF receptor (GITR), and inducible co-stimulator (ICOS), which can stimulate the proliferation and activation of immune cells after activation, improve the TIME, and enhance the efficacy of anti-tumor immunotherapy (52). 4-1BB is a well-studied stimulatory receptor, which interacts with its ligand to provide a second costimulatory signal independent of CD28 signal for T-cell activation, promotes T-cell proliferation and activation, and inhibits activation-induced apoptosis (a major type of programmed cell death of T cells, referred to as AICD), thereby enhancing T-cell immune killing function; moreover, binding of 4-1BB to its ligands can induce the activation of cells, such as monocytes, DCs, etc., and the secretion of corresponding cytokines, which plays a promoting role in immune regulation (53). Therefore, 4-1BB is considered a potential target for enhancing anti-tumor immunity. Currently, it is widely used for the development of targeted antibodies and chimeric antigen receptor (CAR)-T products (54). New drugs for 4-1BB targeted immunotherapy include 4-1BB targeted antibodies ADG106, LVGN6051, and PD-L1/4-1BB-bispecific antibodies ES101 and ATG-101. Recently, researchers have explored several strategies such as intratumoral (IT) administration, design of 4-1BB bispecific antibodies targeting tumor antigens or tumor matrix components, development of proteolytic activation antibodies, and design of tumor-targeting 4-1BB novel antibodies without Fc segments; these are expected to eliminate the key factors causing toxicity while improving the accuracy of antibodies attacking tumors, so that 4-1BB agonists achieve the goal of high efficiency and low toxicity (55, 56). In addition, there are many activating receptors on the surface of NK cells, such as NKp30, NKp44, NKp46, and CD226 of the natural cytotoxicity receptor (NCR) series; these can be designed for immunotherapy so as to target NK cell-activating signals (57). NK cells express human receptor III on their surface for recognition by the Fc region of immunoglobulin G (FcγRIII/CD16), which can bind to the Fc of monoclonal antibodies and trigger antibody-dependent cell-mediated cytotoxicity (ADCC) (58). On the basis of this principle, monoclonal antibodies such as α-CD20, α-GD2, α-Her2, and α-EGFR have been successfully marketed, and various bispecific monoclonal antibodies are being developed. Natural killer group 2D (NKG2D) can improve the cytotoxic activity of NK cells by interacting with its ligands, MHC class I chain-related gene A (MICA) and MICB (59); however, cleavage of MICA and MICB by proteases can block this activation, and inhibition of cleavage of MICA and MICB using monoclonal antibodies can improve the survival rate of mice (60).

#### 2.2.2 Application of Stimulating Cytokines

Cytokines, which are messengers that coordinate cellular interactions and immune system communication, are released by immune and non-immune cells in response to cellular stresses such as infection, inflammation, and tumorigenesis (61). Secreted cytokines can rapidly propagate immune signals in a complex but efficient manner to generate potent and coordinated immune responses to target antigens (61, 62). Therefore, the addition of stimulatory cytokines to the TME can improve immune cell activity and enhance anti-tumor immune responses. IL-2 is a key cytokine that regulates the adaptive and innate immune systems. Intravenous infusion of high-dose recombinant IL-2 induces CR in approximately 12% of patients with melanoma (63)and 7% of patients with renal cell cancer (RCC) (64), which tends to be durable. However, the disadvantages of using IL-2 in clinical practice include shorter half-life, high incidence rates of adverse events, and inducible activity of immunosuppressive Tregs (61). In addition, drugs have been designed to selectively activate immunostimulatory low-affinity IL-2Rβγ complexes. Bempegaldes (NKTR-214) is a CD122 agonist that achieves pleiotropic immune activation by preferentially activating the IL-2b receptor; it has a sustained signaling effect, can activate and expand specific anti-tumor effector T cells and NK cells directly in the TME, increases PD-1 expression on the surface of CD8^+^ T immune cells, and facilitates the binding of this protein to a PD-1 inhibitor (65–67). The phase I/II PIVOT-02 study (NCT02983045) (68) evaluated NTRK-214 in combination with nivolumab for the treatment of advanced solid tumors and found that it showed good efficacy, with an overall ORR of 59.5% (22/37) and CRs in seven patients (18.9%) by tumor type and dose cohort. Cellular and gene expression analyses of longitudinal tumor biopsies showed increased infiltration, activation, and cytotoxicity of CD8^+^ T cells, but no enhancement of regulatory T cells (68). Phase II and III trials on NKTR-214 in combination with PD-1 inhibition (NCT03138889 and NCT03635983) or dual CTLA4 and PD-1 inhibition (NCT02983045) are ongoing.

IL-12 can stimulate NK-cell and CD8^+^T-cell proliferation, promote cytotoxic activity (69), and have potent anti-angiogenic effects; thus, it is a potentially important therapeutic cytokine (70). However, similar to IL-2, the short half-life and toxicity of IL-12 hinders its clinical application (71, 72). To avoid toxicity, NHS-IL12 was developed by fusing two IL-12 molecules to antibodies targeting DNA-histone complexes, which are able to selectively deliver cytokines to necrotic tumor regions (73). The IL12-L19L19 fusion consists of IL-12 linked to a tandem Fv fragment derived from mAb L19 that specifically targets tumor tissue (74), and preclinical studies have shown that IL12-L19L19 is more effective than fusion proteins containing intact mAbs such as NHS-IL12 and BC1-IL12 (74). In addition, recent studies have shown that targeting and/or local delivery of IL-12 or combined anti-PD-1/PD-L1 monoclonal antibody therapy could be a promising approach for cancer therapy (75, 76). Nakao et al. (77) found that intratumoral injection of tumor-selective oncolytic bovine poxvirus encoding IL-7 and IL-12 into immunocompetent tumor-bearing mice altered the TME immune status, activated inflammatory immune status in previously poorly immunogenic tumors, had anti-tumor activity in both tumors that are directly injected with this oncolytic bovine poxvirus and those that are not injected distantly with this oncolytic bovine poxvirus, and even led to a complete tumor response. In tumor models that were unresponsive to ICIs combined with anti-PD-1 antibodies or anti-CTLA4 antibodies alone, it was further confirmed that intratumoral injection of an oncolytic bovine poxvirus encoding IL-7 and IL-12 combined with ICI therapy improved anti-tumor activity (77). IL-15 is another cytokine that can promote the generation, proliferation, and activity of anti-tumor NK cells and CD8 ^+^ T cells (61); however, its efficacy as a treatment is limited. The most extensively tested IL-15 superagonist, N-803, has been tested in combination with other immunotherapies (78, 79). Indeed, there is evidence that N-803 in combination with ICIs is efficacious against a range of solid tumor types following progression under prior ICI therapy (80). Therefore, the supplementation or activation of stimulatory cytokines in the TME is potentially a novel anti-tumor therapeutic strategy.

#### 2.2.3 Enhancing Antigen Presentation

T cells specifically recognize tumor-specific or tumor-associated antigenic peptides presented by MHC or HLA class I or class II molecules on the surface of antigen presenting cells (APCs), and naive T cells are activated to prime and kill tumor cells (81). Inhibition of tumor cell antigen presentation processes plays an important role in limiting T-cell immune responses, and immunotherapies against enhanced presentation of TME antigens can be developed. Plinabulin is a novel selective immunoregulatory microtubule binder (SIMBA) that triggers the release of the immune defense protein GEF-H1 to induce the maturation of APCs and DCs, enhance the cross-presentation of tumor antigens to CD8^+^ T cells, and activate effector T cells to target tumor cells. The randomized double-blind clinical phase III study DUBLIN-3 (NCT02504489, ESMO2021, LBA48) evaluated the efficacy of plinabulin used in combination with chemotherapy as a second- or third-line treatment for patients with NSCLC with wild-type EGFR. Intratumoral toll-like receptor 9 (TLR9) agonists can stimulate immature DCs to release large quantities of cytokines (e.g., IFN-α) and facilitate their maturation into APCs by targeting TLR9 on immature plasmacytoid DCs; this promotes the recognition ability of the immune system and increases the infiltration of CD8^+^ T cells in tumors. A multicenter phase 1/2 clinical trial designed to assess the safety and efficacy of intratumoral injection of the TLR9 agonist SD-101 in combination with low-dose radiation therapy in treatment-naive patients with indolent lymphoma showed that almost all patients had tumor shrinkage at the treatment site without treatment-related grade 4 or serious adverse events (82). Moreover, the investigators observed treatment-related increases in CD8^+^ and CD4^+^ effector T cells and decreases in follicular helper T cells and Tregs in TME, which correlated with good clinical outcomes (82). A trial that evaluated the efficacy of TLR9 agonist SD-101 combined with the PD-1 antibody pembrolizumab for the treatment of patients with advanced melanoma reported an overall ORR of 78%, estimated 12-month PFS of 88%, OS of 89%, and tolerability of a good level (83). Combination therapies induce a wide range of immune activations in the TME, including increased infiltration of NK cells, cytotoxic cells, DCs, B cells, and CD8^+^ T cells, which are often associated with enhanced tumor immune responses. The I-SPY2 study (NCT01042379) evaluated the efficacy of adding SD-101 and pembrolizumab to neoadjuvant therapies (paclitaxel, doxorubicin, and cyclophosphamide). Phase Ib clinical trial on another TLR9 agonist, CMP-001, administered alone or in combination with pembrolizumab in patients with anti-PD-L1-resistant malignant melanoma showed that the response rate of the combination therapy was 25% (28/83), with CR in six patients and partial response (PR) in 22 patients. An ongoing multicenter trial (NCT02680184) continues to evaluate the efficacy and safety of intratumoral CMP-001 therapy administered alone or in combination with pembrolizumab in patients with PD-1-resistant melanoma. Currently, FDA has awarded the Fast Track designation for CMP-001 combined with nivolumab plus ipilimumab to support its clinical development as a treatment regimen for patients with unresectable stage III or IV melanoma. Stimulation of the TLR9 pathway of the innate immune system can enhance the adaptive immune response of tumors in the injection and non-injection sites by enhancing the antigen presentation process. This has unmeasurable potential as a combined new target drug for cancer immunotherapy and is worthy of further exploration.

OVs improve immune system recognition of tumor cells by upregulating pathways that are involved in antigen processing and presentation, including increasing MHC class I/II expression on APCs and tumor cells, promoting tumor-associated antigen presentation and recognition, stimulating potential class I IFN responses, and stimulating chemokine production, thereby recruiting T cells and promoting anti-tumor T-cell responses (84). In addition, OVs can also induce TNF and IL-1β, complement responses, and upregulate the expression of selectin in endothelial cells, thus providing a key signal for the infiltration of T cells (84). Irrespective of whether they are natural or acquired and modified or unmodified, OVs are cytotoxic and show tropism toward tumor tissues; they can selectively infect tumor cells, multiply and spread in and between tumor cells, stimulate the immune response of the human body, and amplify immunomodulatory effects (85). Modified OVs are expected to promote T-cell binding to tumor cells using an entirely new approach. In a previous study, a bispecific T-cell engager system (BiTE) was used to bridge T cells (through CD3 specificity) to tumor cells expressing tumor-associated antigens (such as HER2), and then, BiTE was integrated into OVs to construct those with multiple immunoregulatory effects, which can release BiTE into tumor cells and induce T-cell-mediated tumor cell killing (85). A phase II study evaluating the effects of *in situ* OVs (showing adenovirus-mediated herpes simplex virus thymidine kinase expression) + valacyclovir (an antiviral drug) + stereotactic radiotherapy + pembrolizumab therapy (NCT03004183) in patients with triple-negative breast cancer (TNBC) is ongoing. Therapeutic tumor vaccines act on the innate immune system to present tumor-specific antigenic peptides to T cells through APCs, which enhances cytotoxic T lymphocyte (CTL) activation and mediates immune recognition and killing responses to cancer cells (86). Sipuleucel-T is a DC-based cancer vaccine therapy approved for the treatment of advanced prostate cancer (87). The Atalante-1 study used OSE-2101 (ESMO2021. LBA47), a tumor vaccine targeting the HLA-A2^+^-restricted new epitope of five tumor associated antigens (TAAs) commonly associated with lung cancer, for the treatment of patients with HLA-A2^+^ NSCLC showing secondary immune resistance; it represents the first phase III randomized controlled study on lung cancer in the world to obtain positive results. Multiple studies have used breast cancer vaccines, including the PVX-410 vaccine (targeting the overexpressed XBP1 and CD138 peptides of TNBC) (NCT03362060, NCT02826434), folate receptor α vaccine (NCT03012100), and neoantigen vaccine, administered alone or in combination with PD-1/L1 inhibitors for the adjuvant treatment of TNBC or for the treatment of metastatic disease (88). Relevant ongoing clinical trials include a randomized phase I study on neoantigen vaccine ± durvalumab treatment (NCT03199040) and a randomized phase II study on nab-paclitaxel + durvalumab ± neoantigen vaccine treatment (NCT03606967). These advances highlight the clinical application of tumor vaccines in cancer therapy.

#### 2.2.4 Application of Immune Effector Cells

Other immunotherapeutic strategies that are currently being explored to target the TME include adoptive cell therapy (CAR-T cells and TIL therapy). Recently, two types of genetically modified T cells have been developed, namely, CAR-T cells and TCR-engineered T cells, for adoptive transfer, and substantial progress has been made in the treatment of malignant tumors (89). CAR-T technology has entered the developmental stage of the fifth generation. CAR-T cells were precisely constructed using CRISPR gene editing technology to prepare allogeneic CAR-T cells (90). To date, the US FDA approved five CAR-T cell therapeutics according to clinical guidelines. More than 200 studies on CAR-T cells have been conducted using clinical data from the Google website. CT041 is a humanized autologous CAR-T cell drug candidate that was developed in China. It is currently the only CAR-T cell immunotherapy in the world that targets the gastro-specific membrane protein CLDN18.2 (91), has been approved by the US FDA and China FDA (CFDA), and is undergoing clinical trials. It is the first solid tumor CAR-T product to be qualified as a priority drug (PRIME) by the European Medicines Agency (EMA). CAR-T cell therapy for solid tumors is limited by the lack of tumor-restricted and homogeneously expressed tumor antigens, and the combination of CAR-T with other therapies may solve this bottleneck. Tumor cells were infected with vaccinia virus, an OV, coding CD19t that expressed *de novo* CD19 on the cell surface before virus-mediated tumor lysis; it promotes the targeting of tumor cells by co-cultured CD19-CAR-T cells, thereby inducing the secretion of cytokines, showing potent cytolytic activity against infected tumors, confirming the OV19t promoted tumor control by CD19-CARt cells in several mouse tumor models (92). The other approach involves the enhancement of CAR-T function in solid tumors by enhancing donor cells with a chimeric receptor booster vaccine *in vivo*. Amphipathic CAR-T ligands (amph-ligands) have been designed to be transported to the lymph nodes and to decorate the surface of APCs after injection, which lead to the initiation of CAR-T cells in the native lymph node microenvironment (93). Amph-ligand enhancement triggers massive expansion of CAR-T cells, increases the versatility of donor cells, and enhances anti-tumor efficacy in a variety of immunocompetent mouse tumor models, thus supporting the application of this simple non-human leukocyte antigen-restricted method of enhancing CAR-T function to existing CAR-T designs (93).

Recently, researchers have attempted to engineer immune cells that are less immunogenic and have good tumor-killing activity, such as NK cells, which represent a cell type that has the potential to replace T cells and to be used for universal immune cell therapy. CAR-NK cell therapy can not only specifically recognize antigen-expressing tumors through CAR but also eliminate tumors through NK receptor-dependent mechanisms (e.g., ADCC), and lysis occurs in antigen-negative tumors as well (94). Most CAR-NK cell therapies, including those targeting hematologic targets (e.g., CD19, CD20, and CD138) (95–97) and solid tumors (e.g., HER2, GD2, PSCA, and EGFRvIII), have been evaluated for efficacy in tumor xenograft (PDX) models (98–100). Studies show that 68% and 32% of CAR-NK cell therapies are undergoing phase I and phase II clinical studies, respectively. No CAR-NK cell therapy has entered phase III clinical studies. With regard to the use of anti-CD19 IL-15-secreting CAR-NK cells for the treatment of patients with B-cell lymphoma and chronic lymphocytic leukemia (CLL), 8 of 11 (73%) patients with relapsed or refractory CD19-positive cancer achieved a response; 7 of these patients (lymphoma, 4 patients; and CLL, 3 patients) achieved CR, and 1 patient achieved a response with Richter’s transformation component, but had persistent CLL (101). A rapid response was observed within 30 days of infusion at all dose levels, with expansion of the injected CAR-NK cells at low levels that persisted for at least 12 months (101). In addition to CAR-NK, Klichinsky et al. (102) engineered macrophages using CAR-targeting HER2 and evaluated the killing effect of CAR-macrophages (Macs) on tumors using a mouse model; they found that CAR-Macs could effectively kill tumors, reduce lung metastasis of SKOV3 cells, and ultimately prolong OS in the SKOV3 human ovarian cancer mouse model. Studies revealed that the mechanism underlying the key tumor-killing function may be related to CAR-Mac resistance and the reversal of the transformation of TAMs to M2 macrophages (102). Researchers induced iPSC differentiation in pluripotent stem cells to obtain CAR-expressing iPSC-derived macrophages (CAR-iMac) and found that CAR-iMac cells showed antigen-dependent phagocytosis and cytotoxicity toward tumor cells, as well as antigen-dependent polarization effect on M1 macrophages, when co-cultured with lymphoma cells expressing CD19 antigen or ovarian cancer cells expressing mesothelin antigen (103). Subsequently, CAR-iMac cells also showed an antigen-dependent ability to inhibit tumor cell growth in hematological and solid tumor models of mice (103). The above exploration of CAR-immune cells provides new ideas and broad application prospects for cellular immunotherapy of tumors.

In addition to CAR cell therapy, TIL therapy, which obtains TILs that recognize tumor-specific neoantigens from patient tumor tissues, activates and amplifies them, and then transfuses them back into patients for treatment, has been a breakthrough in recent years. The phase II C-144-01 study (104) used lifileucel (LN-144) for the treatment of 66 patients with stage IIIC/IV unresectable melanoma and showed that after 18.7 months of follow-up, the mDOR was not achieved; an ORR of 36% (2 CR and 22 PR), a disease control rate (DCR) of 80%, and a superior effect in the primary refractory subset of anti-PD-1 or PD-L1 therapy were observed. The FDA has granted orphan drug designation to another novel TIL therapy, ITIL-168, for the treatment of stage IIB-IV melanoma. The US FDA granted breakthrough therapy designation to the candidate therapy LN-145 for the treatment of recurrent, metastatic, or persistent cervical cancer that worsens during or after chemotherapy. A phase II study on LN-145 (NCT04111510) autologous TIL infusion therapy and a multicenter phase II InnovaTIL-01 study on lifileucel for melanoma are currently ongoing. IOV-COM-202 (NCT03645928) is a multicenter, multi-cohort, phase II clinical trial on TIL therapy (LN-144, LN-145, and LN-145-S1) administered alone or in combination with PD-1 monoclonal antibody for the treatment of advanced solid tumors (105). Data from the NSCLC cohort published in 2021 showed that the combination group achieved an ORR of 21.4% and a DCR of 64.3%; this group did not reach an mDOR. Among them, one patient with a CR (negative PD-L1 expression) had a response duration of more than 20.7 months (105). This highlights the synergistic and durable efficacy response of TIL therapy combined with PD-1 monoclonal antibody therapy.

**Table 1** provides an overview of the current targets, therapies or drugs and related clinical trials of immunotherapeutic strategies targeting the TME.

**Table 1:** An overview of the current targets, therapies or drugs and related clinical trials of immunotherapeutic strategies targeting the tumor microenvironment (TME).

Immunotherapeutic strategies targeting the TME	Classification	Targets	Therapies/Drugs	Relevant clinical trials
**Therapeutic strategies based on TME inhibitory signaling**	Targeting TME physical barriers	Extracellular matrix Fibroblast activating protein (FAP) Collagen Laminin (FAK)	Inhibition of Rho-kinase and FAK-mediated cell contraction (e.g., Fasudi, H1152, Defactintb) Reduction of matrix components (e.g., FAP gene-editing cells, FAP-vaccine, FAP antibody-nanoparticle, VS-4718, and PEGPH20) Inhibition of matrix cross-linking (e.g., BAPN and miRNA LOX inhibitors) and fibrosis (e.g., pirfenidone, losartan, and tranilast) Improvement of tumor vascular leakage (e.g., bevacizumab) Reduction of the effect of vascular shear stress.	IMpower 150 study LEAP-006 study (NCT03829319) LEAP-007 study (NCT03829332)
	Targeting immune checkpoints	Programmed cell death-1/PD-L1 (PD-1/PD-L1) Lymphocyte-activation gene-3 (LAG-3)T-cell immunoglobulin 3 (TIM-3) T-cell immunoglobulin and ITIM domain (TIGIT) Siglec-15(S15)	Targeting LAG-3: relatlimab (BMS-986916), LAG525 (IMP701), MK-4280, TSR-033, REGN3767, Sym022, INCAGN02385, FS118, BI754111, MGD013, and Eftilagimod alpha (Efti, IMP321, or LAG-3Ig) Targeting TIM-3: anti-TIM-3 or anti-TIM-3/PD-1/L1 bispecific antibodies Targeting TIGIT: Phase III clinical studies on advanced first-line NSCLC treatment in China include the following: MK-7684A-003 trial (PD-L1 ≥ 1%, MK-7684 + Pembrolizumab, NCT04738487), AdvanTIG-302 trial (PD-L1 ≥ 50%, BGB-A1217 + Teicilizumab, NCT04746924), and SKYSCRAPER-01 trial (high PD-L1 expression, Tiragolumab + Atenibizumab, NCT04294810).	Targeting LAG-3: phase II/III RELATIVITY-047 (CA224-047), phase III TACTI-002 trial (NCT03625323), phase II TACTI-003 study, INSIGHT-004 study; Targeting TIM-3: phase I/II clinical trials (NCT03489343, NCT03652077, NCT03099109, NCT02608268, etc.). Targeting TIGIT: phase III clinical studies: MK-7684A-003 trial, AdvanTIG-302 trial, and SKYSCRAPER-01 trial (NCT04294810).
	Targeting immunosuppressive cells	Myeloid-derived suppressor cells (MDSCs) Immunosuppressive macrophages Regulatory cells (Tregs) Tumor-associated macrophage (TAM)	Sitravatinib, an RTK inhibitor that targets TAM receptors (TYRO3, AXL, MerTK) and a variety of similar RTKs,	Phase II study (MRTX-500) Phase III SAPPHIRE study (NCT03906071) Ongoing trails: NCT02978859, NCT02219711, NCT02954991, and NCT03015740
	Targeting inhibitory cytokines	Transforming growth factor-β (TGF-β) Colony-stimulating factor-1 (CSF-1) and CSF-1 receptor (CSF-1R)	M7824 (Bintrafuspalfa), a bifunctional fusion protein targeting PD-L1 and TGF-β CSF-1R antibody: PLX3397, cabiralizumab(FPA008)	Phase Ia/Ib clinical trial (NCT02526017) Phase II clinical trial (NCT03336216)
	Targeting metabolic inhibition signaling	Solute carrier transporter (SLC) family [mainly monocarboxylate transporter (MCT)-1/4, CD36] Acyl coenzyme A cholesterol acyltransferase 1 (ACAT1) Indoleamine 2,3-dioxygenase (IDO)	Targeting MCT-1: AZD3965 CD36-antibody Targeting ACAT1: Avasimibe IDO inhibitors, IO102/IO103 (a vaccine under development that targets IDO and PD-L1)	Targeting MCT-1: clinical trial (NCT01791595) Ref. 51
**Therapeutic strategies based on TME stimulatory signals**	Targeting stimulatory checkpoints	CD27, CD40, OX40, glucocorticoid-induced TNF receptor (GITR), inducible co-stimulator (ICOS), and 4-1BB NKp30, NKp44, NKp46, CD226 of the natural cytotoxicity receptor (NCR) series, and FcγRIII/CD16	4-1BB targeted antibodies ADG106, LVGN6051, and PD-L1/4-1BB-bispecific antibodies ES101 and ATG-101. Monoclonal antibodies targeting FcγRIII/CD16: such as α-CD20, α-GD2, α-Her2, and α-EGFR	
	Application of stimulating cytokines	IL-2/IL-2b receptor IL-12 IL-15	Bempegaldes(NKTR-214): a CD122 agonist, activating the IL-2b receptor The IL12-L19L19 fusion protein Tumor-selective oncolytic bovine poxvirus encoding IL-7 and IL-12 IL-15 superagonist, N-803,	Phase I/II PIVOT-02 study (NCT02983045) Phase II and III trials (NCT03138889 and NCT03635983, NCT02983045)
	Enhancing Antigen Presentation	Function of tumor antigens presentation	Plinabulin, a novel selective immunoregulatory microtubule binder (SIMBA) Toll-like receptor 9 (TLR9) agonists: SD-101, CMP-001 Oncolytic viruses (OVs) and modified OVs: BiTE-integrated OVs, *in situ* OVs (showing adenovirus-mediated herpes simplex virus thymidine kinase expression) Therapeutic tumor vaccines: Sipuleucel-T, OSE-2101, PVX-410 vaccine, folate receptor α vaccine, and neoantigen vaccine	Phase III study DUBLIN-3 (NCT02504489, ESMO2021, LBA48) I-SPY2 study (NCT01042379) Phase II study (NCT03004183) Phase III Atalante-1 study Ongoing clinical trials NCT02680184, NCT03362060, NCT02826434, NCT03012100, NCT03199040, and NCT03606967.
	Application of immune effector cells	Hematologic targets (e.g., CD19, CD20, and CD138) Solid tumors (e.g., HER2, GD2, PSCA, and EGFRvIII) Immune cells	CAR-T cells, CT041 targeting the gastro-specific membrane protein CLDN18.2, CD19-CAR-T cells, CAR-T cell therapy triggered by Amph-ligands CAR-NK cells, anti-CD19 IL-15-secreting CAR-NK cells CAR (targeting HER2)-macrophages (Macs) CAR-expressing iPSC-derived macrophages (CAR-iMac) TILs: lifileucel (LN-144), ITIL-168, LN-145 and LN-145-S1	Phase II C-144-01 study Phase II study on LN-145 (NCT04111510) Phase II InnovaTIL-01 study IOV-COM-202 (NCT03645928)

## 3 Challenges Faced by TIME Research and Solutions

Immunotherapy strategies have a positive therapeutic effect and potentially cure a small number of patients with advanced high-grade tumors, and in most cases, TME blocks the immunotherapeutic effect by dynamic evolution through compensatory feedback mechanisms and induces drug resistance and even tumor progression. At present, the challenges associated with TME therapy include the lack of research models, complexity of TME immune networks, spatiotemporal heterogeneity of the TME, and the impact of systemic immunity.

### 3.1 Complexity of TIME

The TIME is a complex multi-level system, so there is still room for improvement in immunotherapies targeting the TME. By integrating multi-omics techniques, such as multi-parameter flow cytometry, RNA sequencing, protein array, and spatial tissue characterization, key insights into the composition and transcriptome of the most abundant immune cell populations originating from a variety of extracranial tumors in patients with brain metastases have been proposed; this information revealed the complexity of glioma TIME (106). With recent advances in single-cell technology, the large-scale characterization of tumor-infiltrating immune cells at single-cell resolution is of great interest to cancer immunologists. Sun et al. (107), for the first time, conducted a deep and comprehensive dissection of the unique TIME ecosystem of early recurrent HCC using single-cell transcriptome sequencing technology, which is an important step in understanding how the immune microenvironment affects tumor recurrence.

### 3.2 Spatiotemporal Heterogeneity of TIME

The spatially heterogeneous distribution of the TIME poses a challenge for targeted anticancer immunotherapy. A study used multi-omics analysis technology to comprehensively evaluate the immunophenotype and spatial heterogeneity of NSCLC TME by analyzing the spatial characteristics of surgical resection biopsy at multiple sites in the tumor mass of patients and found that the immune microenvironment shows a high degree of spatial heterogeneity, which results in large regional variations in the tumor (108). A systematic overview of TIL profiles in different cancers examined using single-cell technology could reveal the unique mechanisms of immune responses and elucidate differences in responses between different cancer types. However, the TME is dynamically evolving and is characterized by strong plasticity. The team constructed a nanoimmunomodulator-hydrogel superstructural drug delivery system that can remodel TIME *in situ* based on self-assembled oligopeptide hydrogels and biomimetic nanoimmunomodulators, which can cascade remodel the immune microenvironment of tumor killing in a variety of ways to curb the recurrence of postoperative glioblastoma multiforme (GBM) (109). Multi-parameter and multi-locus analyses will facilitate the comprehensive assessment of the heterogeneous characteristics of the immune microenvironment in tumors, whereas dynamic monitoring and effective remodeling of TME immune characteristics are required to effectively improve immunotherapy efficacy.

### 3.3 Systemic Immunity Affects TME Immune Response

In addition, tumor immunity is not limited to the local microenvironment of the tumor but also relies on the assistance of the peripheral immune system. Mass spectrometry analysis of all immune cells in the bone marrow, spleen, blood, lymph nodes, and tumor revealed remodeling of a large number of peripheral immune cells (110). In three different animal models of breast cancer (AT3,4T1, MMTV-PyMT), the proportions of neutrophils, eosinophils, and monocytes increased, which corresponded with a decrease in the proportions of DCs, B cells, and T cells in the TME (110); this indicates that the systemic immune system forms an immune network cycle of continuous communication during tumor development, whereas the integrity of the body’s immune cycle is closely related to the TME immunophenotype and the response to anti-tumor immunotherapy. Therefore, current therapies targeting the TME are associated with many challenges that need to be analyzed comprehensively and to be overcome so that we can improve the accuracy and effectiveness of these strategies.

## 4 Summary and Prospect

**Figure 1:**
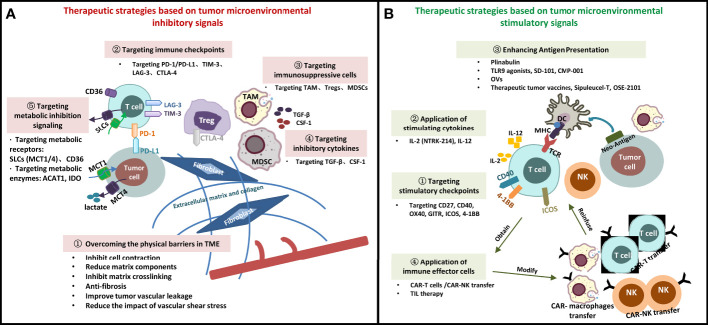
Immunotherapeutic Strategies Targeting Tumor Microenvironment. **(A)** Therapeutic strategies based on tumor microenvironmental inhibitory signals; **(B)** Therapeutic strategies based on tumor microenvironmental stimulatory signals. SLCs, solute carrier transporters; DC, dendritic cell; MDSC, myeloid-derived suppressor cell; TAM, tumor-associated macrophage; Treg, regulatory cell; NK, natural killer cell; MHC, major histocompatibility complex; TCR, T cell receptor; PD-1, programmed cell death-1; PD-L1, programmed cell death-ligand 1; TIM-3, lymphocyte-activation gene -3; TGF-β, transforming growth factor-β; CSF-1, colony-stimulating factor-1; CTLA-4, cytotoxic T lymphocyte-associated antigen-4; β2M: β2- microglobulin; MCT, monocarboxylate transporter; ACAT1, Acyl coenzyme A cholesterol acyltransferase 1; OVs, oncolytic viruses; TILs, infiltrating lymphocyte cells; CAR, chimeric antigen receptor; TLR, toll-like receptor 9.

Starting from the TME signals, we summarize drug research and development for many new targets, including physical barriers, immune cells and surface molecular receptors, cytokines, and metabolic factors (**Figure 1**). However, it should be noted that the treatment of targeted TME is affected by various factors, such as the systemic immune system (host factors), anti-tumor therapy, and tumors, in such a way that a minor change affects the whole system. With the development of novel technologies, the understanding of mechanisms of immune response in the TME has gradually deepened, which is conducive to the discovery of more effective therapeutic targets and the optimization of immunotherapy regimens. Simultaneously, novel drug delivery platforms, such as the TME-responsive nanomedicine systems, must be constructed on the basis of the local environmental properties of TME, including low nutrition, low pH, tediousness, and ischemia (111, 112). The future trend of precision testing involves continuous breakthroughs in new technologies (genomics technology/liquid biopsy, single-cell protein/RNA analysis, multidimensional combined detection technology), new targets, and new drugs. Moreover, a data sharing culture and resources and repositories that can support data sharing are needed to reduce the time from discovery to practice, and through large sample accumulation, development and application of targeted TME immunotherapeutic strategies, in the future, with the help of cloud computing and artificial intelligence (AI) technology seem promising.

## Author Contributions

RB collected article data and wrote the manuscript, JC reviewed and revised. All authors contributed to the article and approved the submitted version.

## Funding

This work was supported by grants from Jilin Provincial Science and Technology Department (20190303146SF); Jilin Provincial Department of Finance Project (JLSWSRCZX2020-0023); Jilin Province Biotherapeutic Science and Technology Innovation Center Project (20200602032ZP).

## Conflict of Interest

The authors declare that the research was conducted in the absence of any commercial or financial relationships that could be construed as a potential conflict of interest.

## Publisher’s Note

All claims expressed in this article are solely those of the authors and do not necessarily represent those of their affiliated organizations, or those of the publisher, the editors and the reviewers. Any product that may be evaluated in this article, or claim that may be made by its manufacturer, is not guaranteed or endorsed by the publisher.
