# Erratum: Development of immunotherapy strategies targeting tumor microenvironment is fiercely ongoing

**DOI:** 10.3389/fimmu.2022.1004587

**Published:** 2022-08-11

**Authors:** Frontiers Production Office

**Affiliations:** Frontiers Media SA, Lausanne, Switzerland

**Keywords:** cancer, tumor microenvironment, immunotherapy, inhibitory signaling, stimulatory signaling

Due to a production error, there were inaccuracies in the section numbering. The corrected subsection numbering should read as follows:

1. Introduction

2. Immunotheraputic Strategies Targeting the TME

 2.1 Therapeutic Strategies Based on TME Inhibitory Signaling

  2.1.1 Targeting TME Physical Barriers

  2.1.2 Targeting Immune Checkpoints

  2.1.3 Targeting Immunosuppresive Cells

  2.1.4 Targeting Inhibitory Cytokines

  2.1.5 Targeting Metabolic Inhibition Signaling

 2.2 Therapeutic Strategies Based on TME Stimulatory Signals

  2.2.1 Targeting Stimulatory Checkpoints

  2.2.2 Application of Stimulating Cytokines

  2.2.3 Enhancing Antigen Presentation

  2.2.4 Application of Immune Effector cells

3. Challenges Faced by Time Research and Solutions

 3.1 Complexity of TIME

 3.2 Spatiotemporal Heterogenity of TIME

 3.3 Systemic Immunity Affects TME Immune Response

4. Summary and Prospect

The publisher apologizes for this mistake.

The original version of this article has been updated.

